# Osteoclast Activated FoxP3^+^ CD8^+^ T-Cells Suppress Bone Resorption *in vitro*

**DOI:** 10.1371/journal.pone.0038199

**Published:** 2012-06-06

**Authors:** Zachary S. Buchwald, Jennifer R. Kiesel, Richard DiPaolo, Meghana S. Pagadala, Rajeev Aurora

**Affiliations:** Department of Molecular Microbiology and Immunology, Saint Louis University School of Medicine, St. Louis, Missouri, United States of America; Institut national de la santé et de la recherche médicale (INSERM), France

## Abstract

**Background:**

Osteoclasts are the body’s sole bone resorbing cells. Cytokines produced by pro-inflammatory effector T-cells (T_EFF_) increase bone resorption by osteoclasts. Prolonged exposure to the T_EFF_ produced cytokines leads to bone erosion diseases such as osteoporosis and rheumatoid arthritis. The crosstalk between T-cells and osteoclasts has been termed osteoimmunology. We have previously shown that under non-inflammatory conditions, murine osteoclasts can recruit naïve CD8 T-cells and activate these T-cells to induce CD25 and FoxP3 (Tc_REG_). The activation of CD8 T-cells by osteoclasts also induced the cytokines IL-2, IL-6, IL-10 and IFN-γ. Individually, these cytokines can activate or suppress osteoclast resorption.

**Principal Findings:**

To determine the net effect of Tc_REG_ on osteoclast activity we used a number of *in vitro* assays. We found that Tc_REG_ can potently and directly suppress bone resorption by osteoclasts. Tc_REG_ could suppress osteoclast differentiation and resorption by mature osteoclasts, but did not affect their survival. Additionally, we showed that Tc_REG_ suppress cytoskeletal reorganization in mature osteoclasts. Whereas induction of Tc_REG_ by osteoclasts is antigen-dependent, suppression of osteoclasts by Tc_REG_ does not require antigen or re-stimulation. We demonstrated that antibody blockade of IL-6, IL-10 or IFN-γ relieved suppression. The suppression did not require direct contact between the Tc_REG_ and osteoclasts.

**Significance:**

We have determined that osteoclast-induced Tc_REG_ can suppress osteoclast activity, forming a negative feedback system. As the CD8 T-cells are activated in the absence of inflammatory signals, these observations suggest that this regulatory loop may play a role in regulating skeletal homeostasis. Our results provide the first documentation of suppression of osteoclast activity by CD8 regulatory T-cells and thus, extend the purview of osteoimmunology.

## Introduction

The skeletal system is dynamically and constantly remodeled throughout life to maintain bone integrity. There are multiple layers of regulation imposed on the skeletal system homeostasis, including physiological levels of phosphate, calcium, hormones, mechanical loading (e.g. Wolff’s law) and energy metabolism (reviewed in [1]). Two cells play a key role in remodeling bone: osteoclasts and osteoblasts. Osteoclasts are large multinucleated cells that are the principal, if not sole, bone resorbing cells in the body. Osteoclasts are derived from the myeloid lineage and therefore may be considered a specialized immune cell. Balancing the function of the osteoclasts are osteoblasts, of mesenchymal origin, which form new bone. Osteoblasts also provide essential signals for, and regulate the differentiation of, the myeloid lineage osteoclast precursors by producing macrophage colony-stimulating factor (M-CSF), receptor activator of NF-κB ligand (RANKL; Tnfsf11) and other co-stimulatory factors in the bone marrow [2], [3], [4], [5].

It has been recognized in the last decade that skeletal homeostasis is dynamically influenced by the immune system. This emerging field, called osteoimmunology [6], arose from observations demonstrating that lymphocyte-derived cytokines, including RANKL, interleukin (IL)-17 and type I and II interferons, are potent mediators of osteoclast function and osteoclastogenesis [7], [8], [9], [10], [11], [12], [13]. Osteoclast activity and numbers are increased by cytokines produced by effector T-cells leading to bone erosion in inflammatory diseases such as rheumatoid arthritis and periodontitis. T-cell produced cytokines also play a critical role in bone cancers, post-menopausal osteoporosis and in Paget’s disease [14], [15], [16], [17].

The immune system also maintains two counterbalancing cell types: the effectors (e.g. T_H_17), which are dominant during the inflammatory phase, and the regulatory T-cells (T_REG_). The transcription factor FoxP3 is a marker of T_REG_ that have the ability to suppress aberrant activation of self-reactive lymphocytes. Loss of FoxP3 function results in fatal autoimmune pathology affecting multiple organs in both humans and mice [18], [19], [20], [21]. Adoptive transfer of T-cells expressing FoxP3 into mice with FoxP3 loss-of-function abolishes the autoimmune pathology [22], [23], [24], [25]. Regulatory T-cells that express FoxP3 also express CD25, the α-chain of the IL-2 receptor. The transfer of CD4 T-cells depleted of the CD25^+^ fraction (∼10%) from a normal adult mouse into a mouse lacking an intact immune system produces autoimmune disease [26]. Conversely, transfer of the CD25^+^ CD4 T-cells from normal mice into T-cell–deficient mice suppressed allergy and prevented graft-versus-host disease after bone marrow transplantation [27]. T_REG_ mediate their regulatory function through a number of mechanisms. First, T_REG_ express anti-inflammatory cytokines including IL-10, TGFβ and IL-35 [28], [29], [30], [31]. Another mechanism of regulation is by cell-cell contact: cytotoxic T-lymphocyte antigen-4 (CTLA-4) expressed on T_REG_ binds with ∼10 fold higher affinity to co-stimulatory B7 molecules on antigen presenting cells (APC) than CD28, and thus prevent APC from activating naïve T-cells [32]. T_REG_ have also been proposed to prevent differentiation of effector T-cells by consuming IL-2, IL-4 and IL-7 required for T-cell activation and polarization [33].

Compared to CD4 T_REG_, the FoxP3^+^ CD8 T-cells (referred to as Tc_REG_ here) have not been extensively studied due in part to their low abundance in lymphoid tissue, and the ability of CD4 T_REG_ to regulate activation of both CD4 and CD8 T-cells [34], [35], [36], [37]. While a few recent studies have indicated that Tc_REG_ may also regulate the immune system [38], [39], [40], their physiological role in immune regulation has not been definitively established. Other regulatory CD8 T-cells that do not express FoxP3 have also been observed and studied [41].

Here we examined the interaction between osteoclasts and Tc_REG_. We uncovered the osteoclast–CD8 T-cell interaction using a time-series microarray dataset to study osteoclastogenesis. Our data showed that RANKL treatment of bone marrow monocytes coordinately induced a number of chemokines and MHC class I presentation pathway during differentiation [42]. In follow up experiments, we showed that the osteoclast-secreted chemokines preferentially recruited CD8 T-cells. Cross-presentation (i.e. presentation of peptides from extracellular proteins on MHC class I) of antigens to CD8 T-cells induced IFN-γ, IL-6 and IL-2 only in the presence of antigen. Antigen presentation by osteoclasts has also been subsequently demonstrated for human osteoclasts derived from peripheral blood mononuclear cells [43]. The priming of naïve CD8 T-cells by osteoclasts induced FoxP3, CD25, CTLA-4, RANKL, and CD122 [44]. The osteoclast-primed FoxP3^+^ CD8 T-cells were able to suppress proliferation of naïve responder CD8 T-cells by DCs and thus have a potential regulatory T-cell activity [44]. Interestingly, the Tc_REG_ express IFN-γ and CTLA-4, which inhibit osteoclast activity [45], [46], [47], [48], [49], [50], while RANKL increases osteoclast activity. IL-6 directly inhibits osteoclast activity but can induce RANKL expression in osteoblasts to increase osteoclast function [14], [51]. Thus, these T-cell produced cytokines could potentially activate or suppress osteoclast activity. We therefore tested if Tc_REG_ could regulate osteoclast resorption activity.

## Results

### Induction of Tc_REG_ by Mature Osteoclasts is Antigen-dependent

**Figure 1:**
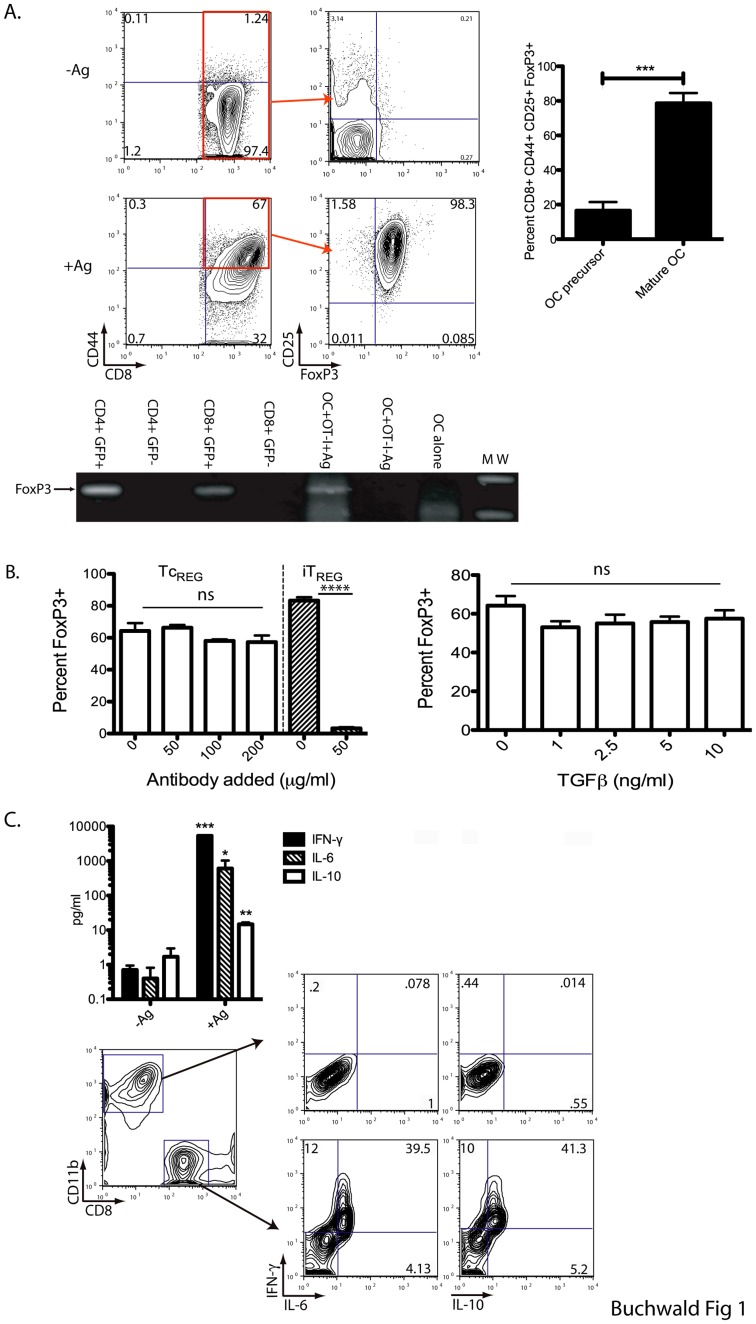
Osteoclast-induced TcREG produce IL-10, IL-6, and IFN-γ. Mature osteoclasts (day 4) or osteoclast precursors were cultured with no Ag or OVA protein and then used to prime CD8 T-cells from an OT-I mouse. **A**. T-cells were collected at 48 h following initiation of co-culture. T-cells were stained for CD44, CD25 and FoxP3 and then analyzed by flow cytometry. T-cells co-cultured with osteoclasts in the absence of antigen do not express FoxP3^+^ or CD25 (top); FoxP3 and CD25 were induced in the presence of antigen as shown in the representative flow plot. The expression of FoxP3 was confirmed by reverse-transcription of RNA isolated from the co-culture and subsequent PCR of cDNA. GFP sorted cells from FoxP3^eGFP^ reporter mice were used as controls. Only mature (day 4) osteoclasts supported the generation of Tc_REG_ (right panel). **B**. Anti-mouse TGFβ was added to co-cultures at the dose indicated (left). Addition of recombinant murine TGFβ1 to co-cultures of CD8 T-cells and osteoclasts at concentration indicated (right). The percent of input T-cells converted to FoxP3^+^ are plotted in both panels. No statistically significant effect was observed on Tc_REG_ induction with either the addition of neutralizing antibody or recombinant TGFβ. **C**. Media was collected and cytokine quantitated by multiplexed ELISA. After 48 h of co-culture, cells were treated with Golgi stop and PMA plus ionomycin for 6 h. The cells were permeabilized, stained and evaluated for cytokine production by flow cytometry. While the CD11b^+^ osteoclasts were negative for all cytokines, the CD8^+^ T-cells stained triple positive for IL-10, IL-6, and IFN-γ. Statistical significance was assessed by non-parametric paired T test: *: P<0.05, **: P<0.01, ***: P<0.001 and ****: P<0.0001.

We first show (Fig. 1A) that mature osteoclasts can induce FoxP3 and CD25 in ovalbumin (OVA)-specific OT-I CD8 T-cells only in the presence of antigen. The induction of FoxP3 in the presence of antigen was assayed by both multicolor flow cytometry (top panels) and by reverse transcription (RT) of RNA isolated from the co-culture, followed by PCR (middle panel). As controls, we isolated RNA from purified GFP positive and negative CD4 and CD8 T-cells from the transgenic FoxP3^eGFP^ reporter mouse [52], [53]. These reporter mice co-express the enhanced green fluorescent protein (eGFP) and transcription factor FoxP3 under control of the endogenous promoter. We show that only mature osteoclasts (incubated in the presence of RANKL for 4 days) have the ability to induce FoxP3 as the osteoclast precursors (bone marrow derived monocytes) could not induce the regulatory phenotype in CD8 T-cells.

TGFβ1 can induce regulatory T-cells in the periphery and *in* *vitro* [24], [54], [55], [56]. Avian osteoclasts have been reported to express the latent form of TGFβ [57]. To assess if murine osteoclasts express TGFβ that could induce Tc_REG_, we measured the expression of TGFβ by quantitative real-time PCR (qRT-PCR). The relative expression (2^ΔΔCt^ with respect to β-actin) of TGFβ1 in osteoclasts (+M-CSF, +RANKL) over osteoclast precursors (+MCSF, -RANKL) was determined to be 5.5±0.8 fold. The relative expression decreased to 0.9±0.6 fold (about the same as in the precursors) in the presence of Tc_REG_, indicating that Tc_REG_ suppress TGFβ expression by osteoclasts. However, these results do not address the role of TGFβ in Tc_REG_ induction. Furthermore, as bone contains significant levels of TGFβ that is released by the action of osteoclasts [58], [59], [60], [61], and the effect of TGFβ can be dose-dependent, we added increasing amounts of TGFβ to the osteoclast and OT-I co-cultures to test its effect on the induction of Tc_REG_. While adding an antibody that neutralizes all three forms of TGFβ at 50 µg/ml efficiently blocked the induction of CD4 T_REG_, the antibody had no effect, at any dose tested, on the numbers of Tc_REG_ induced (Fig. 1B left). Addition of recombinant murine TGFβ1 also had no statistically significant effect on the induction of Tc_REG_ by osteoclasts at any concentration of TGFβ tested (Fig. 1B right). Taken together these results indicate that while osteoclasts express TGFβ1, the cytokine is not required for induction of Tc_REG_ by osteoclasts; furthermore, in the presence of Tc_REG_, TGFβ1 mRNA levels decreased in osteoclasts.

Consistent with our previous observations [44], the induction of FoxP3 coincides with production of the cytokines IFN-γ, IL-6, IL-10, again only in the presence of OVA (Fig. 1C top). All of the cytokines measured by ELISA were found to be expressed by the CD8 T-cells as measured by intracellular staining and flow cytometry and not by osteoclasts (Fig. 1C bottom). These results show that only mature osteoclasts have the ability to induce CD25, FoxP3 and the cytokines IFN-γ, IL-6 and IL-10 in T-cells in an antigen-dependent manner. Finally, we did not detect any change in the levels of CD80, MHC class I surface expression, or detectable levels of MHC class II, on the osteoclasts after 48 hour of co-culture with CD8 T-cells or Tc_REG_ (data not shown).

### Tc_REG_ Suppress Osteoclast Resorption Activity *in vitro*

To directly test if osteoclast-induced Tc_REG_ affect resorption activity, we co-cultured purified ovalbumin-specific transgenic OT-I Tc_REG_ with osteoclasts (1∶1 ratio). The osteoclasts were grown on hydroxyapatite-coated slides or wells to measure resorption activity, rather than bone slices to avoid any confounding effects of TGFβ present in the bone. The data is presented as the total area resorbed and the number of pits in the presence of T-cells relative to osteoclast only cultures. The co-cultures were performed with either “*in-situ*” produced Tc_REG_, or Tc_REG_ induced by osteoclasts plated on tissue-culture plates, then transferred to osteoclasts growing on hydroxyapatite-coated slides.

**Figure 2:**
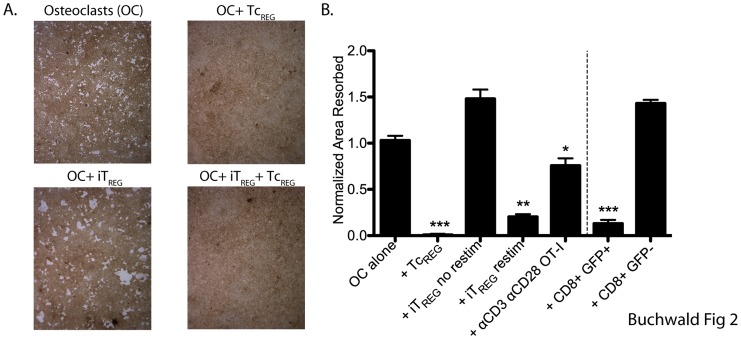
TcREG inhibit osteoclast resorption. A. Osteoclasts (day 3) lifted and plated on hydroxyapatite-coated plates. OT-I Tc_REG_ or OT-II TGFβ-induced FoxP3^+^ CD4 T-cells (iT_REG_) generated in separate dishes were added next day. The co-cultures were re-fed every two days. After seven days, the wells were treated with bleach, photographed, and total pit area was quantified (results in panel B). Representative images from five replicates are shown in panel **A**. No pitting was observed in the presence of Tc_REG_ (top right). Larger pits were observed in the presence of iT_REG_ (bottom left), but Tc_REG_ were dominant suppressors (bottom right). **B**. Tc_REG_ suppressed pitting on hydroxyapatite plates without re-stimulation. In contrast, iT_REG_ could only suppress after re-stimulation (see methods for details). IFN-γ producing OT-I T-cells activated by anti-CD3 and anti-CD28 in the presence of IL-2 could partially suppress pitting by osteoclasts. Activated GFP^+^ CD8 T-cells purified by cell sorting from FoxP3^eGFP^ reporter mice could also suppress osteoclast pitting, while conventional (GFP^−^) CD8 had no affect on pitting. Statistical significance of area resorbed was assessed by Wilcoxon test: *: P<0.05, **: P<0.01 and ***: P<0.001 relative to osteoclast alone wells.

Our results (Fig. 2A, B) show that the *in situ* produced Tc_REG_ suppressed osteoclast resorption activity. We also tested TGFβ-induced OT-II (CD4) T_REG_ (iT_REG_) in the co-culture assay. Whereas, in the presence of the induced OT-II T_REG_ osteoclast resorption activity modestly increased (1.5-fold in area resorbed), re-stimulation of the T_REG_ with anti-CD3 + anti-CD28 was required to observe suppression. In contrast, the osteoclast-induced Tc_REG_ could suppress in the absence of re-stimulation. We also tested if CD8 T-cells when activated by anti-CD3 and anti-CD28 in the presence of IL-2 could suppress resorption by osteoclasts. While CD8 T-cells activated in this manner produce significant levels of IFN-γ, they were only able to partially suppress (∼25% suppression) pitting by osteoclasts. Finally, we tested if *in* *vivo* generated Tc_REG_ could suppress osteoclast activity. Tc_REG_ were purified from the bone marrow space by cell sorting to >95% purity, then co-cultured with osteoclasts (at a ratio of 3 osteoclasts to 1 GFP^+^ T-cell) on hydroxyapatite-coated plates. The CD8^+^ GFP^+^ Tc_REG_ population was able to efficiently suppress osteoclast resorption activity, whereas the CD8^+^ GFP^−^ population did not (Fig. 2B).

Our results show that the osteoclast-induced Tc_REG_, whether produced by co-culture, or isolated from a mouse, have the ability to suppress osteoclast activity. In experiments shown below, we address the mechanism by which the Tc_REG_ mediate this suppression of osteoclasts.

### Tc_REG_ Suppress Osteoclast Differentiation but not Survival

**Figure 3:**
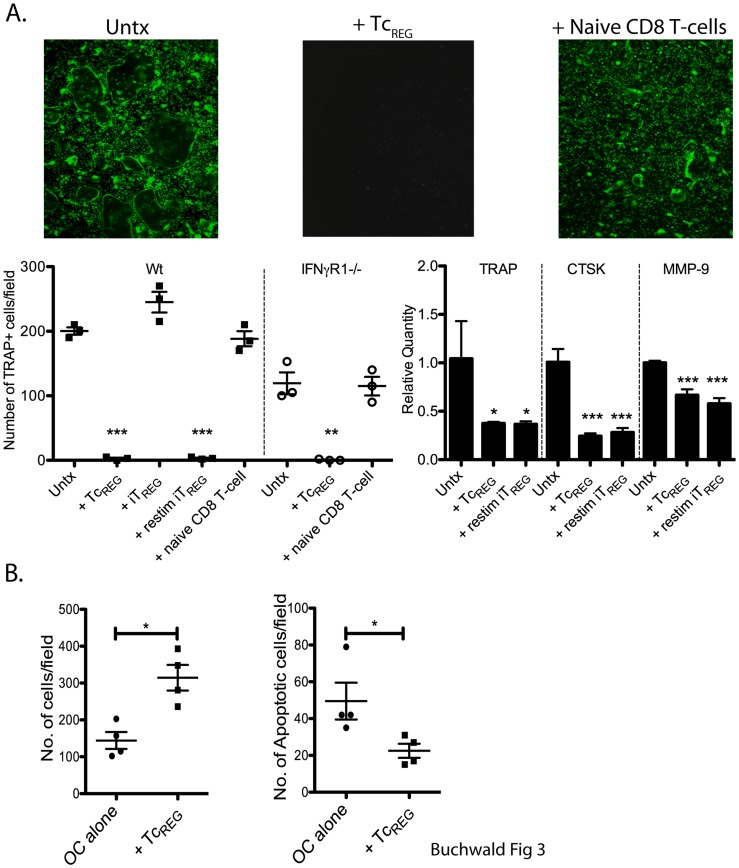
Osteoclast-induced Tc_REG_ inhibit osteoclast differentiation, but not survival. **A**. Osteoclast precursor cells (day 0) were cultured alone, with pre-differentiated Tc_REG_, iT_REG_, anti-mouse CD3/CD28 re-stimulated iT_REG_, or with naïve T-cells in the presence of GST-RANKL. After four days, non-adherent cells were removed by aspiration and remaining cells were stained with a fluorescent TRAP substrate ELF-97. Representative images from four experiments are shown in top panel, and quantitated cells counts are shown below. To test if suppression of osteoclastogenesis was mediated solely by IFN-γ, bone marrow cells from IFNγR1^−/−^ mice were used. The results of TRAP staining were confirmed by quantitative real-time PCR using primers for Acp5 (TRAP), Cathepsin K (CTSK) and matrix metalloprotease-9 (MMP-9). β-actin expression was used for normalization. **B**. Osteoclasts, cultured in the absence or presence of Tc_REG_ for five days were counted after TRAP staining with ELF-97. To test for increased apoptosis adherent cells were stained with Annexin V. Each data point is average of three wells per experiment. Statistical significance was assessed by non-parametric paired T test: *: P<0.05, **: P<0.01 and ***: P<0.001 by comparison to untreated (Untx) osteoclast wells.

IFN-γ blocks osteoclast differentiation, while RANKL promotes differentiation and survival of the osteoclasts. As the pitting assays described above require 7 to 10 days, one mechanism by which Tc_REG_ could mediate a loss of pitting would be to suppress osteoclast differentiation. To test for the effect of Tc_REG_ on osteoclastogenesis, we cultured bone marrow cells with M-CSF for three days, and then removed non-adherent cells to enrich for osteoclast precursors; RANKL was then added with Tc_REG_ (generated on independent wells with mature osteoclasts). After four days of co-culturing we visualized and enumerated the osteoclasts using the fluorescent substrate ELF-97 for tartrate resistant acid phosphatase (TRAP), a marker of osteoclasts [62]. The results show that no TRAP positive cells were induced by RANKL (top middle panel Fig. 3A) in the presence of Tc_REG_. In contrast, many large TRAP positive osteoclasts were observed in the absence of T-cells (top left), and in the presence of naïve CD8 T-cells (upper right). Sato et al. [8] have previously shown that TGFβ-induced T_REG_ cannot suppress osteoclastogenesis, where as Zaiss et al. [49] obtained the opposite results. Our results (Fig. 3A) show that iT_REG_ cannot suppress osteoclastogenesis unless they are restimulated with anti-CD3 and anti-CD28. To test if IFN-γ is solely responsible for suppression of osteoclast differentiation, we used bone marrow from IFN-γ receptor knockout (IFNγR1^−/−^) mice. We found that Tc_REG_ could efficiently suppress osteoclast differentiation of IFNγR1^−/−^ precursors, indicating that additional cytokines play a role in mediating suppression of osteoclastogenesis. Finally, we confirmed the suppression using quantitative real-time PCR of three osteoclast markers (Fig. 3A bottom right). These marker genes were chosen because they are expressed in mature osteoclasts and absent in T-cells.

Next, we considered if Tc_REG_ could control osteoclast numbers, and hence function, by regulating their survival or lifespan. For instance, IFN-γ can induce Fas ligand expression in osteoclasts, in the presence of TNFα to induce apoptosis [63]. In other cell types, TGFβ and IL-18 have been shown to induce Fas ligand [64], [65]. As a measure of the effect of Tc_REG_ on osteoclast survival, we used the numbers of TRAP and Annexin-V positive osteoclast after five days of co-culturing. The results show that Tc_REG_ do not decrease the survival of osteoclasts, as there was a slight increase in the number of osteoclasts (Fig. 3B left panel), and a slight decrease in the number of Annexin-V positive osteoclasts (Fig. 3B right panel) in the presence of Tc_REG_. The results of these experiments indicate that Tc_REG_ suppress the differentiation of the osteoclasts but do not significantly affect their survival.

### Tc_REG_ Suppress Mature Osteoclasts by Preventing Cytoskeletal Reorganization

**Figure 4:**
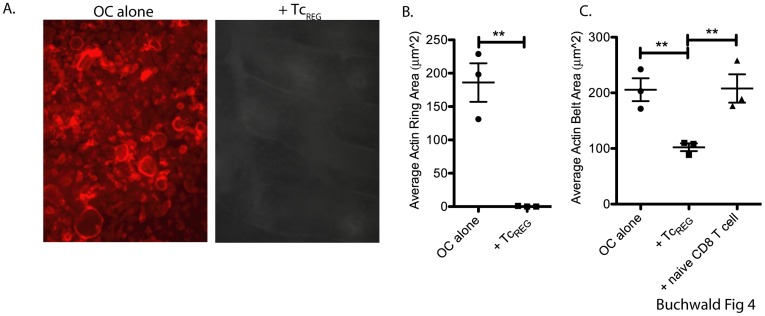
Tc_REG_ suppress mature osteoclasts by effecting the cytoskeletal reorganization: Osteoclasts were cultured either alone or with Tc_REG_ on bovine bone chips for 24 hrs. T-cells were then removed, and osteoclasts were stained with phalloidin-Texas Red to visualize actin rings. Representative images are shown in panel **A**. Quantitation of three independent experiments is shown in panel **B**. Panel **C** is quantitation of phalloidin staining of osteoclasts plated on tissue culture treated dishes. Statistical significance of actin ring area was assessed by non-parametric paired T test: **: P<0.01 in comparison to osteoclast alone.

Osteoclasts must adhere to the bone surface and migrate along it to resorb [66]; cytoskeletal reorganization is critical for this process [67], [68]. Therefore, we stained for actin rings in osteoclasts in the presence and absence of Tc_REG_. Tc_REG_ were cultured with mature osteoclasts seeded on bone, or on plastic for 24 h. T-cells were then removed and the actin rings were visualized using flour-conjugated phalloidin. In the presence of Tc_REG_, the actin ring was either eliminated on bone (Fig. 4A and B), or (the actin belt) was reduced in size on plastic (Fig. 4C).

These results indicate that Tc_REG_ can act directly on mature osteoclasts and prevent the formation of sealing zones. Our data indicates that Tc_REG_ and restimulated iT_REG_ can efficiently block osteoclastogenesis, but not their survival. As osteoclasts express MHC class I but not class II [44] it is possible that co-culturing Tc_REG_ in the pitting assay with the osteoclasts re-stimulates the Tc_REG_ but not the iT_REG_. Therefore, in the next set of experiments we tested if suppression of osteoclast activity requires antigen presentation by osteoclasts. We also tested if direct cell contact between Tc_REG_ and osteoclasts is needed to mediate suppression.

### Suppression of Osteoclasts Activity is Mediated by Tc_REG_ Secreted Cytokines

**Figure 5:**
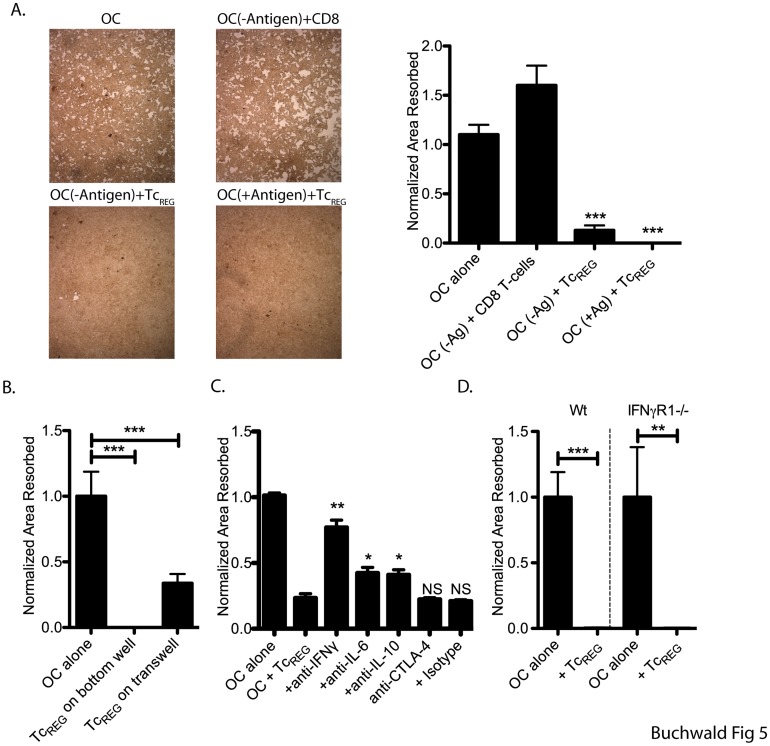
Tc_REG_ inhibit osteoclast activity in an antigen- and contact-independent manner by secreted cytokines. **A**. Osteoclasts were seeded on hydroxyapatite-coated plates and allowed to adhere overnight. The osteoclasts were pulsed with SIINFEKL peptide (+Antigen) or a control FLAG peptide (-Antigen). OT-I Tc_REG_ or naïve CD8 T-cells were added and co-cultured for 7 days. Cells were re-fed with medium containing M-CSF and RANKL every 2 to 3 days. The plates were then treated with bleach solution, washed, dried and photographed. Representative photomicrographs are shown on the left. Quantitation from four experiments of three wells each is shown on the left. **B**. Tc_REG_ were added to top-insert of transwell (0.45-µ membrane) separated from the osteoclast plated on 24-well Corning Osteo-Assay plate. Osteoclast activity was determined by quantifying total pit area resorbed following 10 days of co-incubation. **C**. In a culture of both osteoclast and Tc_REG_ on Corning Osteo-Assay plates, neutralizing antibodies against IL-10 (25 µg/ml), IFN-γ (50 µg/ml), IL-6 (20 µg/ml) or CTLA-4 (10 µg/ml) were added to determine their impact on osteoclast activity. The cells were co-cultured for 10 additional days, and re-fed with media containing M-CSF, RANKL every three days. Re-feed media with antibodies were added on days 3 and 6. **D**. Pitting assay as described in Panel C were conducted in parallel using osteoclasts from either wild type (WT) or IFNγR1^−/−^ mice. Statistical significance of area resorbed was assessed by non-parametric paired T test: *: P<0.05, **: P<0.01 and ***: P<0.001 in comparison to osteoclast alone wells.

To test if the regulation of osteoclast activity was antigen-dependent, OT-I FoxP3^+^ T-cells were cultured with osteoclasts in the presence or absence of OVA peptide. As shown in Fig. 5A, Tc_REG_ could suppress osteoclasts to similar levels in the presence or absence of antigen presentation. Therefore, we next tested if direct contact between osteoclasts and Tc_REG_ was required to mediate suppression. Tc_REG_ were able to suppress osteoclast activity to a significant level when separated by a membrane with a 0.45 μ pore (Fig. 5B). These results indicated that the secreted cytokines were, to a large extent, responsible for the suppressive activity.

To identify the specific cytokines responsible for osteoclast suppression, pre-differentiated Tc_REG_ were incubated with mature osteoclasts in the presence of neutralizing antibodies. Blockade of IFN-γ showed the most significant reduction in osteoclast inhibition while neutralizing IL-10 and IL-6 also relieved suppression (Fig. 5C). CTLA-4 blockade showed no effect confirming that the secreted cytokines IL-6, IL-10, and IFN-γ all contribute to osteoclast suppression. Osteoclasts express F_C_ receptors (FcR), and there could be a concern about confounding effects from the added antibody. Although, we used rat anti-mouse antibodies to minimize the likelihood of mouse FcR from binding to the rat antibodies, a possibility remains. Second, the active moiety of some of the cytokines, notably IFN-γ is a dimer [69] it is possible that immune complex formation could confound the results. To directly address these issues we added duck Hepatitis B virus (dHBV) polymerase to our co-culture, and titrated mouse-anti dHBV polymerase antibody (20 to 70 µg/ml). We observed no effect on suppression (data not shown), indicating that the antibodies, and potential immune complex formation did not confound the assay.

To confirm the results of antibody neutralization we generated osteoclasts from IFNγR1 knockout mice. We found that Tc_REG_ generated from OT-I mice could suppress IFNγR1^−/−^osteoclast pitting to a similar extent as wild-type osteoclasts (Fig. 5D). Taken together, our data document that Tc_REG_, once generated can suppress osteoclast resorption directly through secreted cytokines. Altering the concentration by neutralizing antibody against all secreted cytokines expressed by Tc_REG_ relieved suppression, while blockade of Tc_REG_ expressed CTLA-4 had no measurable effect of suppression.

## Discussion

Many studies have now established that under inflammatory conditions, T-cell produced cytokines increase osteoclast activity leading to bone erosion [70], [71], [72]. This close relationship between the skeletal and immune systems is illustrated in diseases such as autoimmune rheumatoid arthritis and periodontitis. The crosstalk between the immune and skeletal system has been termed osteoimmunology. Osteoimmunology emerged from the early pioneering work of Suda and colleagues (reviewed in [72]) and the demonstration that RANKL, a critical mediator of osteoclasts, is expressed on T-cells [6]. Here we examined the regulation of osteoclasts by FoxP3^+^ CD8 T-cells (Tc_REG_). Although Tc_REG_ have been documented in humans and mice [18], [22], [39], [73], [74], [75], [76], [77], [78], [79], [80], [81], [82], [83], [84], [85], they have not been studied extensively, in part due to their low abundance (0.2 to 2% of CD8 T-cells) in lymphoid organs. In comparison, the well-studied CD4 regulatory T-cells, T_REG_, comprise 5–12% of CD4 T-cell in the spleen. The Tc_REG_ and the T_REG_ have overlapping and distinct functions. Both cells express CD25 and the transcription factor, FoxP3 a marker of the regulatory T-cells [34], [86], [87].

Based on the mediator cytokines produced by the Tc_REG_, in this work we examined if the osteoclast induce Tc_REG_ could control osteoclast activity. Our results show that the osteoclast-induced Tc_REG_ secreted cytokines (Fig. 1) that could suppress resorption by osteoclasts (Fig. 2). Induction of Tc_REG_ by osteoclasts required antigen (Fig. 1), through cross presentation by osteoclasts [44]. Once activated, Tc_REG_, unlike the T_REG_, do not require restimulation to suppress osteoclasts (Fig. 2). To examine the mechanisms by which suppression is mediated, we measured the effect of the Tc_REG_ on differentiation of osteoclasts. Our data shows that Tc_REG_ and iT_REG_ block osteoclastogenesis (Fig. 3A). The Tc_REG_ did not affect the survival of osteoclasts (Fig. 3B), but Tc_REG_ could directly act on mature osteoclasts to suppress actin ring formation (and actin belt formation; Fig. 4). To elaborate on the requirement that Tc_REG_ do not require restimulation, we showed that Tc_REG_ could suppress osteoclast activity in the absence of antigen (Fig. 5A) and when separated from the osteoclasts by a membrane with a 0.45 µ pore (Fig. 5B) indicating the secreted cytokines mediate the suppression. To indentify the cytokine(s), we used neutralizing antibodies against the secreted cytokines and found that altering the levels of any of the three cytokine relieved the suppression of osteoclast activity (Fig. 5C). Indeed, Tc_REG_ could effectively suppress pitting of IFNγR1^−/−^ osteoclasts (Fig. 5D). Although the role of Tc_REG_ in controlling the immune system is still being defined [88], our results provide a new physiological role for the Tc_REG_: the inhibition of osteoclast activity under homeostatic conditions.

Regulatory T-cells use a number of mechanisms to suppress the immune system as noted above, however only a subset of the mechanisms may be expressed in a given situation. In this context, we note that the osteoclast-induced Tc_REG_ produce IL-6 and IFN-γ, molecules that would normally be considered pro-inflammatory, but previous studies have shown they can affect osteoclast activity [89]. Based on a number of recent studies that demonstrate a remarkable plasticity in T_REG_ responses [90], [91], we speculate that this may be a general phenomenon for regulatory T-cells: the method of activation and their location may allow them to express specific effector functions, to regulate cells at hand. This phenomenon may underlie the apparent multiple types of T_REG_ observed [34], [87].

Summarizing, we demonstrate that FoxP3^+^ CD8 T-cells can suppress osteoclast resorptive function and thus provide a novel control function for regulatory T-cells beyond regulation of the immune system. The ability of osteoclasts to induce Tc_REG_ and the ability of Tc_REG_ to subsequently regulate osteoclast function establishes a bi-directional regulatory loop between these two cells in the bone marrow. Notably, the regulatory loop does not require the presence, *in vitro*, of pro-inflammatory cytokines. Indeed, our ability to isolate functional Tc_REG_ from mice, in the absence of any inflammatory disease (Fig. 2B), indicates that these cells have a role in maintaining skeletal homeostasis *in vivo.* In contrast to CD4 T_REG_, the Tc_REG_ may have a more specialized and local function. The ability of osteoclasts to induce Tc_REG_ and their ability to suppress without restimulation may provide an explanation for the low levels of Tc_REG_ found *in vivo*: the system is rapidly inducible, so a large reservoir of Tc_REG_ is not needed. The induction of the Tc_REG_ by osteoclasts that suppress osteoclasts would be self-limiting and lead to small number of Tc_REG_. Osteoclasts remodel bone as part of basic multicellular unit (BMU) that includes osteoblasts and other cells [92], [93]. The rapid induction of Tc_REG_ also suggests that the local concentration of the Tc_REG_-produced cytokines is likely to be high, thus requiring a small number of Tc_REG_. This notion is consistent with our observation that Tc_REG_ suppressed osteoclast slightly less well when separated in a transwell. Finally, in contrast to CD4 T_REG_ cells, which require MHC class II for activation, regulatory CD8 T-cells can be activated by any MHC class I expressing cells. All cells (except red blood cells) express MHC class I and therefore could activate Tc_REG_, an abundance of Tc_REG_ could suppress host response to pathogens.

Why does the immune system regulate osteoclasts? We suggest two possibilities: First, regulatory T-cells have evolved to suppress the immune system. As osteoclasts are derived from myeloid cells, they retain the ability to respond to immune signals. Just as cytokines produced by effector T-cells activate osteoclast activity in inflammatory bone erosion diseases, the cytokines produced by Tc_REG_ suppress osteoclasts. In addition to ontogeny, a functional linkage may also exist. The bone marrow is the primary site of hematopoiesis. Stromal cells line, provide essential support and form a specialized sealed compartment (“niche”) for the hematopoetic stem cells (HSC) [94], [95], [96], [97]. It has been documented that osteoclast activity modulates the egress of the hematopoetic precursors (HPC) from the niches [98], [99]. We hypothesize that the immune system may increase osteoclast activity through production of effector T-cells (T_EFF_). The T_EFF_ secreted cytokines increase osteoclast activity during inflammation to replenish lost immune cells and thus increase circulating hematopoetic precursors (HPC). To maintain balance or restore homeostasis after inflammation, Tc_REG_ may be used to suppress osteoclast activity. For example, it is conceivable that the Tc_REG_ evolved to provide an elegant sensor for T-cell lymphopenia. A reduction in Tc_REG_ numbers may lead to an increase in bone resorption, and the subsequent increase in HPC mobilization. More studies are needed to explore the consequences of this bidirectional regulation for both the bone and autoimmune regulation, and to identify the sensors that mediate this regulation.

## Materials and Methods

Mice: Five-week-old male C57BL/6 mice were purchased from Jackson Labs or used from in-house breeding colony. A breeder trio of FoxP3eGFP reporter mice on a C57BL/6 background were purchased from Jackson Labs, and bred in-house for these experiments. OT-I/Rag^−/−^ and OT-II/Rag^−/−^ mice were purchased from Taconic. All animals were maintained in the Department of Comparative Medicine, Saint Louis University School of Medicine in accordance with institutional and Public Health Service Guidelines. Saint Louis University School of Medicine Institutional Animal Care and Use Committee approved all procedures performed on mice (Protocol 2072). Purchased animals were allowed to acclimate for at least one week, prior to use.

### Generation of Osteoclasts

Osteoclast precursors were isolated as previously described [42], [44]. Briefly, the mice were sacrificed by CO_2_ asphyxiation and the long bones harvested. The caps of the bones were removed and bone marrow cells were flushed with 0.05% collagenase (Worthington) in α-minimum essential medium (αMEM, Invitrogen). The cell population was filtered through a 40 µ cell strainer, pelleted, resuspended and maintained in αMEM growth medium (αMEM supplemented with 10% heat-inactivated fetal bovine serum [Invitrogen]), penicillin-streptomycin-glutamine (Invitrogen) and recombinant murine M-CSF (Peprotech) at 20 ng/ml). Osteoclasts were generated by addition of recombinant murine GST-RANKL (gift of Steven Teitelbaum, Washington University in St. Louis) to a final concentration of 50 ng/ml. M-CSF and GST-RANKL were added every 48 to 72 h. Cells were harvested by a 15 m treatment with Versene (Gibco).

### Isolation of Splenocytes

Single cell suspensions of spleens were prepared in PBS +1% FBS by grinding with a sterile syringe plunger and dispersed by pipetting, then filtering through a 40 µ cell strainer. For co-culture experiments, OT-II CD4 or OT-I CD8 T-cells were prepared by first enriching for T-cells using Pan-T-cell beads then purified by negative selection using appropriate magnetic beads (Miltenyi). The resulting T-cells were routinely >97% pure when stained with anti-CD3, anti-CD4 and anti-CD8 antibody.

### Generation of Tc_REG_

Day 3 osteoclasts cultured in 20 ng/ml M-CSF and 50 ng/ml GST-RANKL, were seeded at 5×10^5^ cells/ml/well in the presence of 5 µM OVA (A-5503; Sigma-Aldrich) in 24-well tissue culture-treated plates (Corning). After overnight incubation, medium was removed and (adherent) cells were washed with pre-warmed (37°C) medium. 2.5×10^5^ freshly harvested splenic OT-I transgenic T cells purified by negative selection were added in 2 mls of complete T-cell media (RPMI, 10% heat-inactivated FBS (ΔFBS; HyClone), penicillin-streptomycin-glutamine (HyClone), non-essential amino acids, sodium pyruvate, HEPES, and 55 µM β-mercaptoethanol. Following 48 h co-culture, T-cell aliquots were removed and stained intracellularly to assay for FoxP3 expression. TGFβ -induced T_REG_ were generated as previously described [24]. Briefly, CD4 T-cells were purified from an OT-II spleen by magnetic beads, and stimulated with plate-bound anti-CD3 (1 µg/ml) and anti-CD28 (2 µg/ml) in the presence of IL-2 and rhTGF-β1 (5 ng/ml). After 48 h of stimulation, the cells were expanded in IL-2 (100 U/ml) containing media for 48 hours. The iT_REG_ were used directly, or in some experiments, restimulated with plate bound anti-CD3/anti-CD28 (as above) for an additional 48 hours in the absence of rhTGFβ.

### Antibodies and Fluorescence Activated Cells Sorting (FACS)

Anti-mouse antibodies for FACS were: PE-conjugated anti-mouse CD8a (clone 53-6.7; BD Pharmingen), AF700-conjugated anti-mouse CD44 (clone IM7; BD Pharmingen), e450-conjugated anti-mouse FoxP3 (clone FJK-16s, eBioscience), APC-conjugated anti-mouse IFN-γ (XMG1.2, eBioscience), FITC-conjugated anti-mouse IL-10 (JES5-16E3, eBioscience) and FITC-conjugated anti-mouse IL-6 (MP5-20F3, eBioscience), and PE-Cy7-conjugated anti-mouse CD11b (M1/70, eBioscience). Cells were blocked with anti-mouse FcgRIII/IIR (BD Pharmingen) for 10 m and then stained for 45 m on ice with fluorophore-conjugated antibody. Stained cells were pelleted, washed, fixed with 3% paraformaldehyde and analyzed on LSR instrument with CellQuest (BD Biosciences) software. FACS data analyses were performed with FlowJo (Tree Star).

Abs for cytokine neutralization were: anti-mouse IL-10 (clone JES5-2A5, eBioscience), anti-mouse IFN-γ (clone R4-6A2, eBiosciences), anti-mouse IL-6 (clone MP5-20F3, eBiosciences), clone 1D11.16 [100] that recognizes all three forms of mammalian TGFβ, and anti-mouse CTLA-4 (clone 63828, R&D Systems).

### Cytokine Profiling by Multiplexed ELISA

Cytokine quantitation (IL-6, IL10, and IFN-γ) was performed using multiplexed ELISA (Millipore/Linco Research) per the vendor’s protocol on a Luminex-100. The data was analyzed using three-point logistic fitting in Microsoft Excel.

### Cytokine Staining

For osteoclast staining, Golgi Stop (BD Biosciences) was added to T-cell/osteoclast co-cultures at 4 µl/(6×10^6^) cells and incubated for 6 h. Osteoclasts were collected using ice-cold PBS and vigorous pipetting. For T-cell staining, 50 ng/ml phorbol 12-myristate 13-acetate and 1 µg/ml ionomycin were added to T-cell/osteoclast cultures for initial 1 h incubation. Golgi Stop (Invitrogen) was then added, and cultured for 3 h. T-cells were then removed from the plate using pre-warmed PBS. Cells were pelleted (5 min at 400×g) and resuspended in PBS plus 1% ΔFBS. Cells were blocked with rat anti-mouse FcγIII/IIR mAb (BD Biosciences Pharmingen) in 1% ΔFBS in PBS and stained for 45 min at 4°C with fluorophore-conjugated Ab, washed, fixed with Fix Buffer (4% Paraformaldehyde (Electron Microscopy Sciences), 0.01% Tween-20 (Sigma) in PBS), washed, permeabilized with Perm Buffer (0.5% BSA (Sigma), 0.1% Triton-X100 (Sigma) in PBS), washed, stained overnight at RT, washed and then analyzed using an LSR II instrument with CellQuest (BD Biosciences) software. Analysis was performed using FlowJo software (version 8.73; Tree Star).

### Differentiation Assay

Freshly harvested osteoclast precursor cells were cultured with 20 ng/ml M-CSF for 3 days on a 15 cm Petri dish (Corning). Cells were removed with Versene and seeded at 8.5×10^4^ cells/well on a 96 well plate with M-CSF to allow BM precursor to adhere. The following day (day 0), GST-RANKL was added to the osteoclast precursor cell culture in the presence or absence of pre-activated Tc_REG_. T-cells were washed off (day 5) and the adherent cells were assayed for tartrate-resistant acid phosphatase activity using a fluorescent substrate, ELF-97 (Invitrogen), in accordance with the manufacturer’s protocol.

**Table 1:** Primers used for RT-PCR.

Gene	Primer sequence	Annealing Temperature (°C)
FoxP3	F: 5′-CCCACAAGCCAGGCTGATCCCR: 5′-AGAGGCAGGCTGGATAACGGCA	58
CTSK	F: 5′-GGACCCATCTCTGTGTCCATR: 5′-CCGAGCCAAGAGAGCATATC	57
TRAP (Acp5)	F: 5′-TTCCAGGAGACCTTTGAGGAR: 5′-GGTAGTAAGGGCTGGGGAAG	57
MMP-9	F: 5′-CATTCGCGTGGATAAGGAGTR: 5′-CACTGCAGGAGGTCGTAGGT	57
TGFβ	F: 5′- GGTGGACCGCAACAACGCCATR: 5′-TGGGGGTCAGCAGCCGGTTA	58
IDO	F: 5′-ACTGTGTCCTGGCAAACTGGAAGR: 5′-AAGCTGCGATTTCCACCAATAGAG	58
β-actin	F: AAGAGCTATGAGCTGCCTGAR: TACGGATGTCAACGTCACAC	58

### Survival Assay

On day 0, mature osteoclast were plated at 8.5×10^4^ cells/well on a 96 well plate alone or with pre-differentiated Tc_REG_. Day 5, non-adherent cells were removed and remaining cells were counted and stained with FITC-conjugated Annexin-V (Biotium).

### Resorption Assays

For in-situ differentiated Tc_REG_, on day 0, mature osteoclast (5×10^5^) were seeded on 24-well hydroxyapatite coated plates (Corning or BD Biosciences). Day 1, osteoclast were pulsed with SIINFEKL peptide for 4 h, washed, and then naïve OT-I T-cells were added. M-CSF and GST-RANKL were added every 48 h. Day 10, cells were removed with 10% bleach and pit area was photographed and quantified using NIH ImageJ.

For pre-differentiated Tc_REG_, day 0, osteoclasts were seeded as above. Day 1 osteoclasts were pulsed with SIINFEKL peptide or no antigen for 4 h, washed, and then pre-differentiated Tc_REG_ were added. M-CSF and GST-RANKL were added every 48 h. On day 7, cells were removed with 10% bleach and pit area was photographed and quantified using NIH ImageJ.

For transwell assays, osteoclasts were plated as described above. Cells were allowed to adhere overnight, and media was replaced with 700 µl of complete T-cell media containing M-CSF and GST-RANKL in the bottom well. 5×10^5^ Tc_REG_ were added to top insert in 300 µl. Cells were re-fed every 48 h with M-CSF and RANKL. Following 10 days of incubation, bottom wells were treated with bleach to remove cells, and pit area was quantified, as above.

### Purification of Tc_REG_ from FoxP3^eGFP^ Reporter Mice

To purify activated Tc_REG_ we activated osteoclasts *in vivo* by injecting the FoxP3^eGFP^ mice with RANKL (1 mg/kg intraperitoneally). Two doses of RANKL were given intraperitoneally on consecutive days; this protocol is modified from [101], where three doses were administered. The mice were sacrificed 50 hours post first RANKL dose and bone marrow cells harvested as described in osteoclast generation section above. Magnetic beads (Miltenyi) were used to positively select CD8 T-cells. The eluted cells were stained with V450-conjugated CD3 (clone 17A2; eBioscience) and PE-conjugated CD8a antibodies and further purified by cell sorting on Aria instrument with FACS Diva software (BD Biosciences).

### Antibody Neutralization Assay

Immature day two osteoclasts were lifted and 8.5×10^4^ cells were seeded on 96-well hydroxyapatite coated plates (Corning). After overnight incubation, pre-differentiated Tc_REG_ (8.5×10^4^) were added with neutralizing antibodies. M-CSF and GST-RANKL were added every 72 h. After additional six days, cells were removed with 10% hypochlorite, photographed and pit area was quantified using NIH ImageJ. The lowest concentration of antibody (see legend Fig. 5C) that gave maximum number of pits was determined from an antibody titration.

### Actin Ring Assay

Mature osteoclast were plated at 3×10^3^ cell/well on bovine bone slides or on plastic in a 48 well plate. Following overnight adherence, Tc_REG_ or naïve OT-I T-cells were added at a 1∶1 T-cell to osteoclast ratio. After a 24 h co-incubation, the non-adherent cells were removed; adherent cells were fixed (4% Paraformaldehyde (EMS), 0.2% Triton-X100 (Sigma) in PBS) for 10 minutes, washed thrice with PBS and then stained with phalloidin-Texas Red (Biotium) for 15 min. The cells were photographed and actin ring size was quantified using NIH ImageJ software.

### RT-PCR and Quantitative Real-time PCR

Total RNA was purified from cell lysates following manufacturers instructions (Agilent technologies). For FoxP3, cDNA was generated (Invitrogen) and subsequently PCR amplified for 45 cycles. The reaction contained 2.5 mM MgCl2, 0.2 mM dNTPs. qRT-PCR for CTSK, TRAP and MMP-9 transcripts were performed on ABI7500 instrument using a one-step (Invitrogen) SYBR green kit for 40 cycles using input 1 ng total RNA. The data is expressed as 2^ΔΔCt^ (relative quantity or RQ) by normalizing to β-actin expression between samples. The primer sequences used for analyses of RNA expression span at least one intron, are shown in Table 1.

### Statistical Analysis

All statistical analyses were performed with GraphPad Prism (version 5.0c). Wilcoxon or Student’s T test were used for determining statistical significance for osteoclast alone and osteoclast plus treatment as indicated in the Figure legends or in the methods described above.
